# Sclerostin activity plays a key role in the negative effect of glucocorticoid signaling on osteoblast function in mice

**DOI:** 10.1038/boneres.2017.13

**Published:** 2017-05-09

**Authors:** Eric E Beier, Tzong-Jen Sheu, Emily A Resseguie, Masahiko Takahata, Hani A Awad, Deborah A Cory-Slechta, J Edward Puzas

**Affiliations:** 1 Department of Environmental Medicine, University of Rochester, School of Medicine and Dentistry, Rochester, NY, USA; 2 Department of Environmental and Occupational Medicine, Rutgers University, Piscataway, NJ, USA; 3 Center for Musculoskeletal Research, University of Rochester, School of Medicine and Dentistry, Rochester, NY, USA

## Abstract

Stress during prenatal development is correlated with detrimental cognitive and behavioral outcomes in offspring. However, the long-term impact of prenatal stress (PS) and disrupted glucocorticoid signaling on bone mass and strength is not understood. In contrast, the detrimental effect of lead (Pb) on skeletal health is well documented. As stress and Pb act on common biological targets via glucocorticoid signaling pathways and co-occur in the environment, this study first sought to assess the combined effect of stress and Pb on bone quality in association with alterations in glucocorticoid signaling. Bone parameters were evaluated using microCT, histomorphometry, and strength determination in 8-month-old male mouse offspring subjected to PS on gestational days 16 and 17, lifetime Pb exposure (100 p.p.m. Pb in drinking water), or to both. Pb reduced trabecular bone mass and, when combined with PS, Pb unmasked an exaggerated decrement in bone mass and tensile strength. Next, to characterize a mechanism of glucocorticoid effect on bone, prednisolone was implanted subcutaneously (controlled-release pellet, 5 mg·kg^−1^ per day) in 5-month-old mice that decreased osteoblastic activity and increased sclerostin and leptin levels. Furthermore, the synthetic glucocorticoid dexamethasone alters the anabolic Wnt signaling pathway. The Wnt pathway inhibitor sclerostin has several glucocorticoid response elements, and dexamethasone administration to osteoblastic cells induces sclerostin expression. Dexamethasone treatment of isolated bone marrow cells decreased bone nodule formation, whereas removal of sclerostin protected against this decrement in mineralization. Collectively, these findings suggest that bone loss associated with steroid-induced osteoporosis is a consequence of sclerostin-mediated restriction of Wnt signaling, which may mechanistically facilitate glucocorticoid toxicity in bone.

## Introduction

Stress and sustained elevation of stress-related hormones like cortisol, which are associated with a variety of disorders and diseases are more prevalent among communities with low socioeconomic status.^1–3^ Elevated stress can occur at any period throughout life. An individual’s allostatic load produced by prolonged or repeated stress challenges creates physiological changes and altered neuroendocrine responses that modify health outcomes.^4^ Importantly, the consequences of sustained stress, especially in the case of prenatal stress (PS), have been reported to produce enduring deleterious effects in offspring^5,6^ and considered a mechanism by which adult diseases are programmed.

Glucocorticoids (GC) are synthesized and secreted by the adrenal glands and have substantial impact on the physiological functioning of several body systems, including metabolism, adaptation to stress events and modulation of host defense, the later being particularly important in consideration of therapeutic administration of GC. The signaling axis of GC consists of the hypothalamic-pituitary-adrenal (HPA) axis and is influenced by a multitude of factors including neuroinflammation, physical stress, circadian rhythm, and negative feedback. Self-attenuation of GC signaling on GC receptors in the hippocampus, hypothalamus and pituitary gland is designed to stabilize the response.

In regard to skeletal health, there are a number of human and non-human primate studies to suggest that PS can effect physical changes in bone growth and birth complications of offspring.^7^ PS is reported to decrease birth weight and gestational length,^8,9^ decrease bone density,^10^ and to increase marrow adiposity later in life.^11,12^ These effects have been attributed to several factors. Chronic PS and prolonged maternal HPA activation can exceed the enzymatic placental barrier, therefore exposing the fetus to increased GC.^13,14^ Elevated levels of maternal GC may disrupt central mediators of growth in the offspring by damaging the fetal HPA axis. It has been demonstrated that GC (PS or dexamethasone given prenatally) have significant consequences for bone later in life, specifically stunting bone growth in male rats preferentially over female rats.^12–15^ Despite the accumulating evidence of long-term consequences of PS on skeletal outcomes and the conceivability of relevant pathways that may influence bone structure, mechanistic understanding of the effects of PS exposure on bone quality is limited.

Lead (Pb) exposure similarly acts on the HPA axis,^16–18^ and has substantial influence on bone density and quality outcomes in rodents and humans.^19–21^ Sustained hypercortisolism by Pb or stress, or as established when given a regimen of GC therapy, produces a skeletal imbalance and precipitous bone loss.^22^ The incidence of secondary osteoporosis with excessive GC is well described and recognized in the literature;^22–24^ however, the mechanisms of GC action are less understood. The primary source of GC-induced bone loss is thought to be direct inhibitory effects on osteoblast activity.^23^

As the Wnt pathway is critical for bone formation,^25^ this study aimed to investigate a mechanism of GC impingement upon Wnt signaling. GC have been associated with inhibition of Wnt signaling in relation to decreased bone formation^26–29^ and increased bone marrow adiposity.^29–32^ An antagonist of Wnt signaling, sclerostin, is secreted by osteocytes and is a potent inhibitor of osteoblastic mineralization.^33^ Several studies have reported a rise in sclerostin (scl) and dickoff-1 (Dkk-1) expression in rodents and cell culture systems following GC treatment.^34–38^ Yet, the evidence for a substantial link in human patients has been discordant with decreased serum sclerostin at one week in GC patients,^39^ and increased serum sclerostin at later time points.^40^ Similar disparities are seen with sclerostin and disease states: increased levels are reported in Cushing’s syndrome patients,^41^ whereas cases of chronic hypercortisolism presented with decreased sclerostin.^42^ Sclerostin activity as a means for heavy metals to facilitate osteoporotic bone loss was first established in animal systems^19,43–45^ and their association was recently revealed in osteoporotic patients.^46^

Based on these concerns, the objective of this study was to assess multivariable exposure to gain new insights into bone-specific effects by both maternal stress and Pb through overlapping GC mechanisms. In addition, we explored a mechanism involving induction of sclerostin as a facilitator of the negative effects of GC on bone quality. Finally, we examined whether the inhibitory effects of GC on bone mineralization were counteracted by removal of sclerostin.

## Materials and methods

### PS model and Pb dosing

The University of Rochester Medical Center IACUC approved all animal studies. Four-week-old female C57BL/6 mice (Charles River) were randomly assigned to receive drinking water containing 0 or 100 p.p.m. Pb acetate (100 Pb). Pb exposure was initiated 2 months prior to breeding to ensure elevated Pb body burden at the time of conception. At proestrus, as determined by vaginal smears, female mice were mated with males (2:1) across two estrous cycles. The presence of vaginal plugs or sperm was considered indicative of pregnancy and deemed gestational day (GD) 1. Pregnant females were then randomly subdivided to a nonstress (NS) or PS condition.

On GD16 and GD17, timed to correspond to the development of key brain regions (hypothalamic nuclei, hippocampus, striatum, frontal cortex),^47,48^ the dams were weighed and those in the PS groups were subjected to a widely employed restraint stress procedure consisting of three 45 min restraint sessions (9:00, 12:00 and 15:00) in plastic cylindrical devices,^49^ a protocol that is verified to elevate corticosterone levels and alter catecholamine levels in the frontal cortex and nucleus accumbens of dams.^18,50^ The NS dams were weighed and subsequently left undisturbed in their home cages. This resulted in four Pb-stress conditions with 6 mice/group: NS (no Pb, no stress), PS (no Pb, PS), 100 Pb+NS (Pb exposure, no stress), 100 Pb+PS (Pb exposure and PS).

From weaning at postnatal day 21, pups were provided with unrestricted access to Pb drinking solutions. At 8 months of age, mice were decapitated, trunk blood was collected, and lumbar vertebrae and legs were harvested from each treatment group.

### Pb bone and blood determination

Blood Pb levels were measured on whole trunk blood using anodic stripping voltammetry on the Lead Care II system (Magellan Diagnostics, Billerica, Massachusetts, USA). Bone Pb levels were quantified using atomic absorption according to previously published methods.^51^ In brief, tibias were dissected and proximal halves were flushed of bone marrow with PBS and incubated in 3% hydrogen peroxide for 20 min. Bones were dried in a 60 °C oven overnight, weighed, and analyzed using atomic absorption spectroscopy.

### Micro-computed tomography

Bone properties were determined from spine and limbs using a 10.5 μm resolution, 55 KVp, 142 μAmp multi-slice cone beam micro-computed tomography (microCT) scanner (VivaCT40 Scanco Medical, Wayne, PA, USA) as described previously.^43^ The following bone properties were generated: bone volume to total volume (BV/TV), trabecular number (Tb.N), trabecular spacing (Tb.Sp), trabecular thickness (Tb.Th), connective density (Conn.D), and structural modality index (SMI). Images were reconstructed to an isotropic voxel size of 15 μm and selected based on mean BV/TV value. For analyses of trabecular bone, we selected a region equivalent to 8% of the femur height, 1.06 mm in total, beginning 0.3 mm from the most proximal aspect of the growth plate. Trabecular bone within the distal femur and proximal tibia was segmented from the cortex using a semi-automated contouring algorithm in the axial plane. We conducted thresholding according to 220 Scanco proprietary guidelines (−1 000/+1 000). For determination of bone properties of spine, we chose the third lumbar vertebrae (LV3) and the entire vertebral body was used, about 2.5 mm in total.

### Biomechanical flexure testing

Isolated bones were stored at −20 °C for up to 1 week, thawed to ambient temperature, and then rehydrated in PBS for 1 h before testing. Destructive three-point bending of right femurs was conducted with the anterior surface in tension at a displacement rate of 3 mm·min^−1^. The span between the two lower supports was set at 8 mm. Maximum load, yield load, material stiffness, and energy absorption data were generated from the load-displacement curve for each specimen (Instron 4465/5500, High Wycombe, United Kingdom).

### Bone histomorphometry and immunostaining

Extracted skeletal elements were fixed in 10% formalin for 4 days. Bones were decalcified for 2 weeks in 14% EDTA. Specimens were then processed, embedded in paraffin, and medial sections were obtained with a HM355S microtome (Thermo Scientific, Waltham, MA, USA) using stainless steel blade at a thickness of three microns. The first three LV (LV1–3) were sectioned longitudinally. Tissue sections were stained with Alcian blue and counterstained with hematoxylin, orange G, and eosin (ABH) for analysis of bone properties. Additional contiguous sections were stained for tartrate-resistant acid phosphatase (TRAP) antigen using naphthol AS-BI phosphate for osteoclasts parameters. Static bone parameters were quantified and expressed according to published methods using Osteomeasure bone analysis software (Osteometrics, Atlanta, GA, USA).^52^ The ROI for tibial trabecular bone was an area (1.23 mm^2^) below the growth plate within the proximal tibial metaphysis and for spine we chose LV3 and the entire vertebral body was used. For intramedullary fat analysis, we counted the number of fat vacuoles of the adipose tissue, which appear optically empty in sections.

Immunohistochemistry for sclerostin was performed. Sections were deparaffinized, washed, and incubated in 10 mmol·L^−1^ citrate buffer, pH 6.0, for 30 min at 80 °C. Sections were incubated overnight at 4 °C in primary antibody. Tissues were washed and then incubated in secondary antibody (Vector Labs, Burlingame, CA, USA) for 30 min. Next, samples were washed and incubated with horseradish peroxide streptavidin for 30 min and developed with 3-amino-9-ethylcarbazole (AEC) chromogen for 5 min. LV were counterstained with hematoxylin, dehydrated through ethanol, and mounted. Quantification of sclerostin positive osteoblastic cells (scl+) was achieved in LV2 by counting the number of brown cells verse blue cells in trabecular bone. We chose 300 μm regions of interest starting 50 μm below the superior growth plate and above the inferior growth plate. Four mice and five sections per mouse were counted.

### GC model treatment and serum ELISA

At 5 months of age, male C57BL/6 mice were given subcutaneous implantation of either placebo or prednisolone pellets (Innovative Research of America, Sarasota, FL, USA). Prednisolone pellets release at a dose of 5 mg·kg^−1^ per day as previously described.^53^ The mice were anesthetized and subsequently sacrificed on 14, 28 or 42 days post implantation with 4 mice per group per time point. Whole blood was collected from vena cava during autopsy and allowed to clot at RT for 20 min, and then centrifuged 25 min at 4 °C. Serum fraction was isolated and stored at −80 °C until analysis. Protein levels of type 1 procollagen (P1NP, Nordic Biosciences Diagnostic), C-terminal telopeptide (CTx-1, RatLaps: Nordic Biosciences, Herlev, Denmark), sclerostin (scl, ALPCO, Salem, NH, USA), DKK-1 (R&D Systems, Minneapolis, MN, USA), and leptin (Enzo Life Sciences, East Farmingdale, NY, USA) were determined according to manufacturers protocols.

### Cell culture and assays

Mouse calvarial osteoblasts were collected from 2-day-old pups as described previously.^54,55^ Mouse calvarial osteoblasts were plated in low-glucose alpha-minimum essential medium (α-MEM, Invitrogen, Carlsbad, CA, USA) with 5% bovine serum plus 50 μg·mL^−1^ ascorbate to confluence for experimentation. Cells were treated with media supplemented with dexamethasone (Dex, Sigma, St. Louis, MO, USA) dissolved in dimethylsulfoxide. Total RNA was isolated using mini columns (Qiagen, Germantown, MD, USA) and reverse transcribed using the iScript cDNA synthesis kit (Bio-Rad, Hercules, CA, USA). Quantitative PCR reactions were carried out using PerfeCTa SYBER green (Quanta Biosciences, Beverly, MA, USA) according to manufacturer’s protocols. Samples were run in triplicate. Quantifying to a standard curve of serial diluted cDNA generated average CT values. Genes of interest were normalized to β-actin expression and vehicle control group was set to 1.

Total protein was collected from treated mouse calvarial osteoblasts and quantified by a BCA protein assay kit (Pierce, Waltham, MA, USA). Twenty-five micrograms of protein was fractionated on 4%–12% Bis-Tris Mini gels. Protein bands were transferred to a PVDF membrane and blocked in 5% milk and then incubated overnight with primary antibody at 4 °C. After washing, membranes were incubated 1 h with secondary antibody and developed with enhanced chemiluminescence (Amersham Biosciences, Little Chalfont, UK). Visualization was done on an Alpha Innotech Fluorochem imaging systems. Antibodies: polyclonal anti-sclerostin and Dkk-1 (R&D Systems), monoclonal anti-active β-catenin (Millipore, Billerica, MA, USA), and polyclonal anti-β-actin (Oncogene Research Products, La Jolla, CA, USA).

### Adipogenesis and osteogenesis assays in bone marrow cells

Bone marrow (BM) cells were isolated from 12-week-old wild-type and sclerostin knockout (SOST-KO) mice^56^ as described previously.^45^ Briefly, femurs and tibias were stripped of soft tissue and epiphysis removed. Marrow cavities were flushed with *α*-MEM by a 22-gauge needle. Cells were spun at 5 000 r·min^−1^ for 5 min. Pellets were resuspended, plated in a 10-cm dish and left undisturbed for 5 days. For adipocyte formation, BM cells were seeded in 12-well plates two days past confluence in high-glucose Dulbecco’s Modified Eagle medium (DMEM), then cultured for 10 days in DMEM plus 10 μg·mL^−1^ insulin and 0.5 mmol·L^−1^ methylisobutylxanthine as described previously.^19^ Cultures were stained with Oil Red O and quantified by dissolving stain in 4% IGEPAL (Sigma) and measuring absorption at 490 nm. For mineral formation, BM cells were seeded in 6-well plates and cultured for 21 days in *α*-MEM plus 10 mmol·L^−1^ β-glycerol phosphate and 50 μg·mL^−1^ ascorbate as described previously.^55^ Cultures were then stained with nitro-blue tetrazolium and 5-bromo-4-chloro-3'-indolyphosphate (NBT/BCIP reagent kit, Life Technologies, Carlsbad, CA, USA) for alkaline phosphatase activity at 10 days and 1% alizarin red S to assess matrix mineralization at 21 days.

### Statistics

Data are presented as mean±s.e.m. Differences between the groups were determined using unpaired student’s *t-*test, while experiments with two independent variables were evaluated using two-way analysis of variance. Dose-dependent effects were determined by one-way analysis of variance, with Bonferroni post-test as criteria for determining differences in means. Statistical significant was given to *P*<0.05.

## Results

### Effects of PS and Pb on bone mass

**Figure 1:**
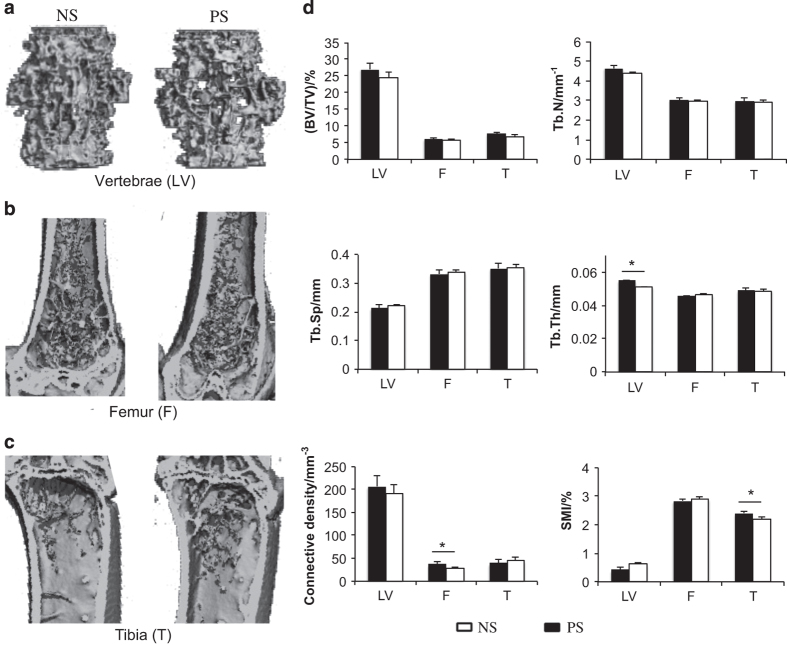
Effect of prenatal stress (PS) on trabecular bone structure. Dams were exposed to stress by restraint chambers on days 16 and 17 of gestation. Bone quality parameters were measured by microCT in 8-month-old male offspring. 3D images (left) are representative transverse sections in (**a**) third lumbar vertebrae (LV), (**b**) femur (F), and (**c**) tibia (T). (**d**) Bone parameter values are presented in the graphs (right). Bar=500 μm. Data are mean±s.e.m. for six mice per group, *P<0.05 for effect of stress. BV/TV, trabecular bone volume/total volume; SMI, structural model index; Tb.N, trabecular number; Tb.Sp, trabecular spacing; Tb.Th, trabecular thickness.

Exposure to PS had a minimal effect on trabecular bone quality in male offspring at 8 months (Figure 1). Trends of depressed bone parameters were found in the spine, femur and tibia (Figure 1a–c), but changes in bone volume did not show significance compared with NS controls. There was evidence of morphological changes in trabeculae as demonstrated by a 6.4% decrease in thickness of spine, 37.9% decrease in connective density of femur, and 9.5% decrease in cylindrical shape of tibia compared with NS controls (Figure 1d). Interestingly, femur length was significantly reduced by 3.2% in PS mice compared with controls (Table 1).

**Figure 2:**
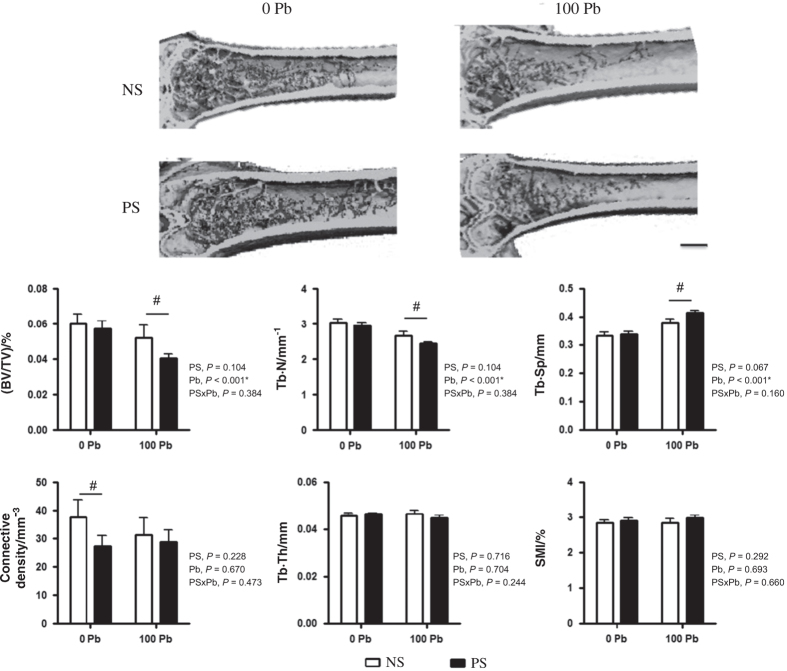
Prenatal stress disposes the skeleton to enhanced deficits in bone quality as a consequence of lead (Pb) exposure. Lifelong treatment of 0 or 100 p.p.m. Pb in drinking water in no stress (NS) and prenatal stress (PS) mice influence trabecular bone properties at 8 months. 3D images (top) are representative microCT sections. Trabecular bone parameters in distal femurs were analyzed (bottom). Modest decrements as a consequence of PS were exacerbated by co-exposure with Pb. Bar=500 μm. Data are mean±s.e.m. for six mice per group, *P<0.05 for effect of stress or Pb, ^#^*P*<0.05 for differences in means using *post-hoc* multiple comparisons.

In addition to PS, we exposed these mice to normal or Pb-treated water to assess whether there was an interactive effect of cortisol and Pb on bone health. Mean blood Pb levels were 7.76 μg·dL^−1^ and bone Pb levels averaged 62.91 ng·mg^−1^ in exposed mice (Table 1). PS had no effect on body Pb distribution. MicroCT analysis of trabecular bone in the femurs of Pb-exposed mice showed significant deleterious changes in bone volume −13.3%, trabecular number −11.9%, and trabecular spacing +1.13-fold compared with water controls (Figure 2). Interestingly, co-exposure amplified the adverse effect in bone parameters, which were greater than either PS or Pb-exposed mice alone (BV/TV −32.9%, Tb.N −19.5%, and Tb.Sp +1.24-fold) compared with water controls. Conn.D in combined-treated mice was 23.4% of water controls, but was not greater than PS mice compared to water controls. There was no difference in trabecular bone thickness or shape, implying that the loss of trabecular number was the primary contributor to the decrease in bone volume.

### Biomechanical strength is compromised in PS- and Pb-exposed mice

Flexure testing was done to assess tensile strength at the femoral midshaft. Statistically significant decreases in material hardness were detected in the bones of PS mice as demonstrated by a 19.3% decrease in stiffness, 27.3% decrease in yield load, and 17.5% decrease in max strength compared with controls (Table 2). Pb-treated mice had lower bone strength as determined by significant decreases in yield load (20.4%) and maximum strength (15.7%) compared with water controls. There were also significant interactive effects of Pb and PS on bone strength parameters, particularly a 26.9% decrease in yield load and a 24.2% decrease in max strength compared with water controls.

### Depressed osteoblastic parameters in PS- and Pb-treated mice

**Figure 3:**
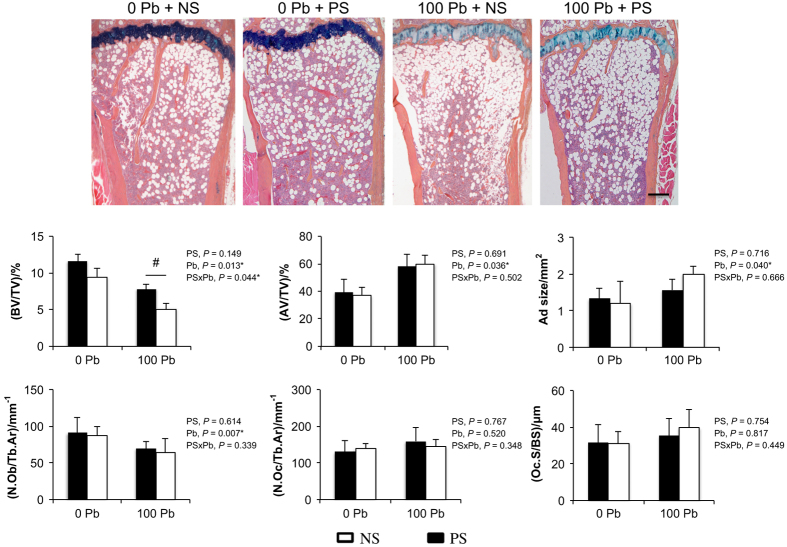
The effects of PS and Pb exposure on adipocyte, osteoblast, and osteoclast formation parameters in trabecular bone. Images are representative alcian blue hematoxylin stains (top) of medial tibial sections from water and Pb-exposed NS and PS mice. Trabecular bone area was measured for histologic parameters and presented in the graphs (bottom). Bar: 100 μm. Data are mean±s.e.m. of four mice per group. *P<0.05 for effect of stress or Pb, ^#^*P*<0.05 for differences in means using *post-hoc* multiple comparisons. Ad size, adipocyte size; AV/TV, adipocyte volume/total volume; BV/TV, trabecular bone volume/total volume; N.Ob/Tb.Ar, osteoblast number/trabecular area; N.Oc/Tb.Ar, osteoclast number/trabecular area; Oc.S/BS, osteoclast surface/bone surface.

To evaluate the relative contributions of cellular processes underlying bone mass, we examined histomorphometric parameters of bone formation and bone resorption. PS produced no significant reduction in trabecular bone volume of the tibia as indicated previously. However in Pb-exposed mice, there was a consistent 32.6% decrease in bone volume (Figure 3). The trabecular volume loss was greater when Pb was combined with PS, as revealed by a 56.0% decrease compared with the water control group. Adipocytes composed 39.3% of the total bone marrow volume in NS mice. Fatty marrow was increased by 1.48-fold in Pb alone, and 1.53-fold in Pb+PS compared with NS mice. Adipocytes were larger in Pb-exposed mice by 1.20-fold, and further increased in Pb+PS mice by 1.51-fold compared with water controls. Measurements of osteoclast number and surface area were found to be unaltered among all groups. However, the number of osteoblasts was reduced 20.6% after exposure to Pb and by 28.8% in Pb+PS mice compared with controls. These data suggest that a primary contributor to the decline in trabecular bone could occur through an effect on the osteoblastic and adipogenic populations.

Serum levels of P1NP and CTx-1 were measured to assess relative activity of osteoblast and osteoclasts in attempt to determine the basis for the synergistic effect of Pb and PS (Table 3). Osteoclast activity marker CTx-1 was not significantly affected by Pb or PS, however, osteoblast activity marker P1NP was significantly reduced in mice exposed to PS and Pb treatment, as well as in combination. Upon examining Wnt antagonist molecules, levels of Dkk-1 were not significantly influence by Pb, PS, or the combination. On the other hand, levels of sclerostin were increased by 1.5-fold in Pb-treated mice, and in PS mice exposed to Pb there was a significant interaction of Pb and PS, as seen with a 2.1-fold induction of sclerostin compared with controls.

### GC treatment produces a decrease in bone formation and elevation of sclerostin

**Figure 4:**
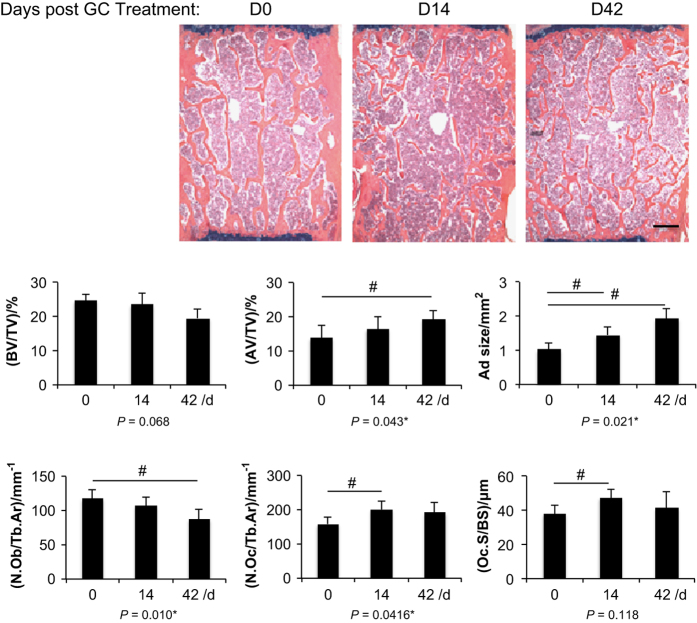
GC treatment decreased osteoblastic parameters while only transiently increased osteoclast parameters. Images are representative alcian blue hematoxylin stains (top) of LV2 from placebo and GC-treated mice at 14 and 42 days post implantation. Trabecular bone area was measured for histologic parameters and presented in the graphs (bottom). Bar: 100 μm. Data are mean±s.e.m. of four mice per group. *P<0.05 for effect of prednisolone, ^#^*P*<0.05 for differences in means using *post-hoc* multiple comparisons. AV/TV, adipocyte volume/total volume; BV/TV, trabecular bone volume/total volume; GC, glucocorticoid; LV, lumbar vertebrae; N.Ob/Tb Ar, osteoblast number/trabecular area; N.Oc/Tb Ar, osteoclast number/trabecular area; Oc.S/BS, osteoclast surface/bone surface.

To study GC signaling and effects on bone formation, prednisolone pellets were implanted in adult mice. GC treatment did not significantly lower bone mass in LV2 (Figure 4). However, there was a significant rise in adipocyte volume over the duration of exposure, including an increase in average adipocyte size. Osteoclastic parameters were considerably increased in number and surface area at 14 days post treatment, but these effects were lessened at 42 days post treatment. GC treatment reduced the amount of osteoblasts reaching a significant level at 42 days post treatment. Bone formation and mineralization apposition rates were considerably reduced 75%–90% by GC administration in trabecular bone reported in a previous study.^53^

**Figure 5:**
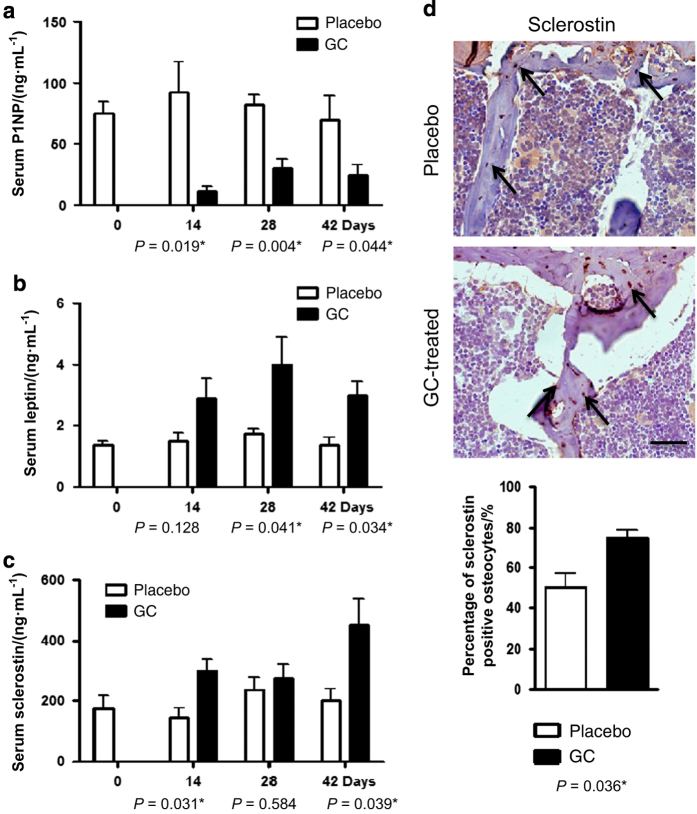
Sclerostin levels are elevated in mice treated with prednisolone. Levels of type 1 procollagen (**a**), leptin (**b**), and sclerostin (**c**) were measured using standard ELISA methods in serum 14, 28, and 42 days after implantation with prednisolone or placebo tablet. (**d**) Images are representative immunohistochemical stains of sclerostin protein in the second lumbar vertebrae after 28 days. Arrows indicate the areas of contrasting sclerostin osteoblastic positive staining. Bar: 500 μm. Data are mean±s.e.m. for four mice per group, *P<0.05 for effect of prednisolone.

Serum P1NP levels remained between 74–92 ng·mL^−1^ throughout the 48-day experimental duration (Figure 5a). At 14 days post implantation, procollagen levels had dropped 88.0% compared with controls. P1NP levels began to recover by 48 days, with a relative 68.4% decrease compared with controls. The presence of leptin ranged from 1.4 to 1.8 ng·mL^−1^ in placebo-treated mice (Figure 5b). Prednisolone increased leptin levels by 1.9-fold at 14 days, with a peak increase of 2.3-fold by day 28 post implantation. Circulating serum sclerostin levels were between 144 and 238 pg·mL^−1^ (Figure 5c). Sclerostin was significantly elevated 2.1-fold on day 28 and 2.3-fold on day 48 following prednisolone treatment compared with placebo controls.

In addition, we determined *in vivo* sclerostin protein expression by immunostaining of vertebrae (Figure 5d). The depicted areas are borders where trabeculae descend from the superior growth plate. Sclerostin expression was observed in osteocytes and mature osteoblasts, but was absent in cartilage cells. There was moderate staining found in the placebo-treated animals, with 48.7% of the osteocytes positive for sclerostin. However, 76.4% of osteocytes were positive with detectable sclerostin protein following implantation of prednisolone, a significant increase of 1.6-fold over controls.

### Effects of dexamethasone on sclerostin and *β*-catenin signaling

**Figure 6:**
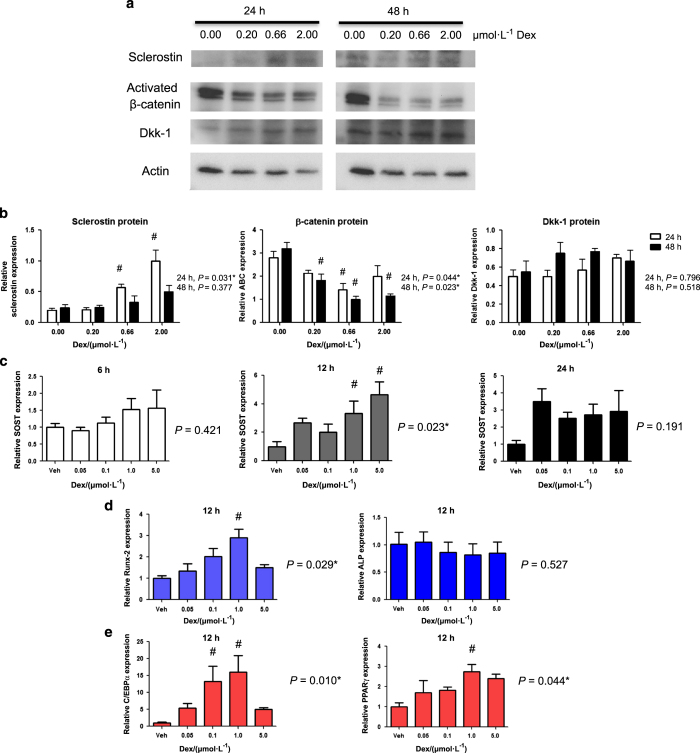
Effect of dexamethasone on Wnt signaling in primary cells. Isolated mouse calvarial osteoblasts were treated with dexamethasone. (**a**) Expression of Wnt signaling proteins was determined by Western blotting after 24 and 48 h. (**b**) Blots of interest were quantified using ImageJ relative to Actin levels. (**c**–**e**) Expression profiles of *SOST*, *Runx2*, *ALP*, *C/EBP-α,* and *PPAR-γ* were assessed over 24 h by quantitative PCR following exposure to dexamethasone. Data are mean±s.e.m. for three trials, *P<0.05 for effect of dexamethasone, ^#^*P*<0.05 for multiple comparisons of means.

The consensus sequence for a GC response element (GRE) is AGAACAnnnTGTTCT. However, studies have shown that variants to that sequence still allow GC binding to DNA. Monomeric GC can bind to DNA half sites,^57^ and the half site TGTTCC has been shown to effectively induce transcription of GC-regulated genes.^58^ There are four such putative GRE half sites within the sclerostin promoter sequence. Following treatment of mouse calvarial osteoblasts with the synthetic GC Dex, sclerostin protein levels (Figure 6a) were increased 4.8-fold by 2.0 μmol·L^−1^ Dex at 24 h (Figure 6b). In comparison, another Wnt signaling pathway antagonist, Dkk-1, displayed no significant elevation by Dex. Increasing corticoid exposure diminished the levels of active β-catenin protein 63.4% by 2.0 μmol·L^−1^ Dex treatment at 48 h.

*SOST* gene expression was dose-dependently elevated by Dex at 12 h, with a peak 4.7-fold elevation with 5 μmol·L^−1^ Dex (Figure 6c). RNA levels remained increased at 24 h, with a 2.9-fold elevation from 1 μmol·L^−1^ Dex treatment. 12 h was determined to be the most appropriate time to measure the effects of subsequent gene expression. Similar to *SOST* levels, *Runx2* expression reached peak expression between 12–24 h with a 2.9-fold significant increase with 1 μmol·L^−1^ Dex (Figure 6e). Dex produced no significant change in *ALP* expression. The effect of GC on *C/EBPα* reached peak elevation at 12 h, as exemplified by a 16.2-fold increase with 1 μmol·L^−1^ Dex (Figure 6d). In addition, there was a 2.7-fold increase in *PPAR-*γ transcript with 1 μmol·L^−1^ Dex compared with controls.

### Effects of dexamethasone on bone nodule formation and adipocyte formation

**Figure 7:**
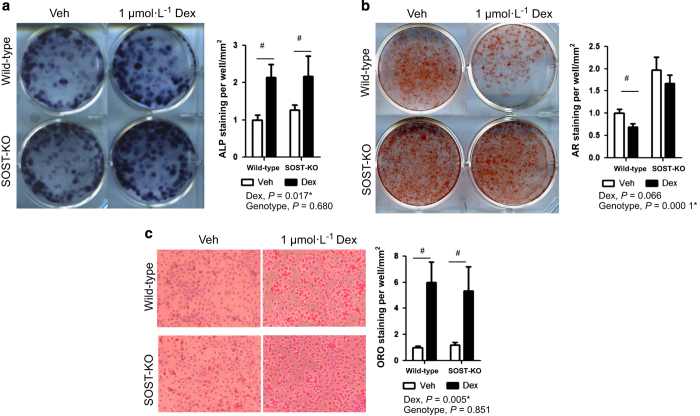
Osteoblasts deficient of sclerostin are resistant to dexamethasone-induced decrease in bone mineralization. Bone nodule formation was assessed by (**a**) alkaline phosphatase and (**b**) alizarin red staining from isolated bone marrow cells of wild-type and SOST-KO mice following 10 and 21 days in osteogenic media and dexamethasone. (**c**) Adipocyte formation was measured by Oil Red O staining after 5 days in adipogenic media plus dexamethasone. Representative stains of cell cultures from each group (left) with quantification (right) are presented. Data are mean±s.e.m. for 3 trials, *P<0.05 for effect of dexamethasone or SOST-KO, ^#^*P*<0.05 for multiple comparisons of means.

Cellular assays were performed to relate gene expression changes as a consequence of Dex to functional outcomes of bone processes. In osteogenic BM cells, 1.0 μmol·L^−1^ Dex treatment produced a 2.1-fold increase in alkaline phosphatase activity, an early stage marker of osteoblast maturation (Figure 7a). No statistical differences were observed in ALP staining of SOST-KO cells exposed to Dex. Despite this increase in ALP staining at 10 days, mineralization of BM cells with Dex treatment had 30.7% less alizarin red staining than wild-type control cultures (Figure 7b). SOST-KO osteoblasts showed more robust bone nodule formation than wild-type osteoblasts by 2.0-fold, and were significantly resistant to the negative effect of Dex. BM cells were induced to form adipocytes under continued presence of Dex. Treatment with 1.0 μmol·L^−1^ Dex produced a 5.9-fold increase in Oil Red-O staining compared to control cultures (Figure 7c). No changes were observed in oil droplet formation of SOST-KO cells exposed to Dex.

## Discussion

Bone loss because of excessive GC signaling is ascribed to decreased osteoblastogenesis and reduced osteoblast function. Yet the effect of GCs on osteoblasts and bone mineralization are convoluted, exhibiting both negative and positive effects depending on the steroid concentration, cell differentiation state, and species. Our findings presented here suggest that disruption of the HPA axis and resulting hypercortisolism resulting from stress due to maternal movement restriction has limited effects on bone mass and trabecular bone, though, noticeable deficits in cortical bone length and strength. And yet, when PS was combined with lifetime Pb exposure, there were enhanced impairments in trabecular bone properties normally associated with Pb treatment alone. Greater decrements were similarly found in the number of osteoblasts and in biomechanical properties in the Pb+PS group, with a significant interaction between the study variables. No new findings were revealed with Pb treatment alone in this study. However, these are the first studies to examine two co-occurring environmental factors, Pb and stress, both that have recognized endocrine disruption and detrimental effects on human bone health.

The precise method of PS effects on bone was not explored here, but we previously reported a prolonged stress response and elevated cortisol levels upon HPA axis disruption by both PS and Pb exposure.^16–18,59^ In particular, developmental Pb exposure permanently alters HPA axis function producing sustained hypercortisolism and altered GC negative feedback. In addition, interruption of bone development has been reported as a consequence of Pb and stress, which in this study, the endocrine changes ultimately produce deficits in bone strength and bone quality later in life. It cannot be discounted that maternal stress and Pb share a number of biological substrates and effects beyond the HPA axis, including deficits in catecholamine systems, behavior responses, and cognition^60–63^ that may also influence skeletal health.

Another objective of this study was to describe the molecular mechanism by which GCs depress bone mineralization. We presented evidence that GCs mediate their effects through sclerostin antagonism of β-catenin signaling. GCs may increase apoptosis of osteoblasts, but also impingement on the Wnt signaling pathway has been shown to inhibit osteoblast differentiation,^64^ a phenomenon that is abrogated with silencing Dkk-1 expression.^65^ The majority of these studies examined changes in Wnt antagonist levels systemically, whereas this report focused on sclerostin modulation in osteoblastic cells and in bone tissue directly, in addition to sclerostin expression peripherally. Sclerostin and prednisolone were elevated in response to dexamethasone in culture within 24 h, both after initial treatment, and sustained levels out to 48 days post treatment.

We posit that elevation of sclerostin by GC signaling is a mechanism for homeostatic bone uncoupling, producing loss of bone mass. Previous reports have linked disruption of Wnt signaling in the prednisolone model as an important mediator of the pathophysiology of GC-induced osteoporosis (GIOP).^27,53,66^ In addition, we observed an increase in leptin protein corresponding with loss of osteoblast activity observed by a decrease in P1NP levels and reduced bone formation.^53^ Leptin acts negatively on bone formation through the sympathetic nervous system via adrenergic signaling on osteoblasts.^67^ Sympathetic neuronal control of bone, through molecules such as GC and subsequently leptin, can differentially regulate cancellous verse compact bone.^67–69^ Therefore it is plausible that disruptions caused by the PS model could alter cortical bone over cancellous bone through leptin and result in decreased bone mineral strength, though further investigations are necessary. The significant decrease in cortical strength in PS mice could not be delineated due to its effect on bone geometry or bone quality. The primary effect of GC is theorized to be suppression of osteoblast activity, and yet, GC temporarily induced osteoclastogenesis. Similar accounts have been documented in both clinical and animal studies that have shown a reduction in the level of osteoprotegerin, an antagonist of receptor activator of NF-*κ*B ligand (RANKL) stimulation of osteoclast differentiation, in the initial stage of GC treatment.^70,71^

Another finding apropos of the sclerostin-mediated inhibition of Wnt signaling is that PS, Pb, and prednisolone have recently been described to divert bone marrow stromal cells to form increasing numbers of adipocytes at the expense of osteoblasts.^19,43,72,73^ These reports indicate that prolonged exposure to GC and Pb shunts the differentiation potential toward an adipogenic lineage through increasing master regulators of adipogenesis, peroxisome proliferator-activated receptor-γ and C/EBP, and decreasing the osteogenic regulator Runx2.^74^ The important pathway gatekeeping this bifurcation is Wnt signaling,^75–77^ in which suppression of Wnt signaling is consistent with promotion of adipocyte formation. In our animal models, Pb produced a profound increase in fatty marrow composition, and we observed increases in adipocytes, the adipokine leptin, and C/EBP as a consequence of GC treatment, which may be indicative of an increase in adipogenesis and body fat stores.^78^

In osteoblast cultures, GCs have been shown to strongly reduce mineral matrix formation.^79,80^ At the molecular level, this is proposed to be a result of apoptotic stimulation, impeded cell cycle progression and downregulation of important osteoblastic genes such as *Runx2*, type 1 collagen, *ALP*, and osteocalcin.^23^ While we found that the apoptotic marker caspase 3 and cell number were not different from controls following Dex treatment at 21 days (data not shown), alizarin red staining was significantly reduced. However, when Dex treatment was administered in sclerostin-deficient cells, osteoblastic mineralization was restored. This result was independent of earlier alterations in osteoblastogenesis as demonstrated by the removal of sclerostin having no relevant alteration in antecedent ALP activity. This points to the fact that sclerostin may play a pertinent role in later stage osteoblasts with GC inhibition of mineralization.

Both GC and Pb have particular significance to human health, as GC therapy is the most common promoter of secondary osteoporosis and Pb is persistent in the environment. The present findings illustrate the effects of GC with Pb exposure through sclerostin-mediated action on bone mineralization. Much work needs to be done to substantiate whether this novel target of intervention may provide a protective effect on bone health and GIOP. The enhancements observed with maternal stress and lifetime Pb exposure highlight the need to examine risk factors with relevant modifiers that co-exist with high frequency in various socioeconomic groups.

## Figures and Tables

**Table 1:** Pb levels and bone stature in 8-month-old male mice

	0 Pb+NS	0 Pb+PS	100 Pb+NS	100 Pb+PS
Blood Pb	0.17±0.19	0.03±0.07	7.86±1.34^a^	7.66±0.88^a^
Bone Pb	0.65±0.07	0.77±0.20	58.67±4.61^a^	67.16±5.36^a^
Bone length/mm	15.49±0.18	14.99±0.12^b^	15.08±0.27	14.96±0.08^b^

Blood: ng Pb·dL^−1^ of peripheral blood; bone, ng Pb·mg^−1^ dry wt of tibial bone; NS, nonstress; Pb, lead; PS, prenatal stress.

Pb determination in soft and mineralized tissues (*n*=4 per group) of stress- and Pb-treated mice. Femurs were measured using a sliding caliper from medial condyle to greater trochanter (*n*=6 per group).

a *P*<0.05 for effect of Pb.

b *P*<0.05 for effect of stress.

**Table 2:** Bone strength properties of femurs in prenatal stress and Pb-treated male mice at 8 months

Treatment	Max stiffness/(*N*·mm)	Yield load/*N*	Max load/*N*	Energy to failure/mJ
0 Pb+NS	113.2±5.5	17.08±0.69	18.15±0.89	3.01±0.23
0 Pb+PS	91.4±3.7^a^	13.41±0.55^a^	14.97±0.74^a^	2.44±0.30
100 Pb+NS	100.0±8.6	13.59±0.54^b^	15.30±0.70^b^	2.43±0.21
100 Pb+PS	91.7±8.6^a^	12.48±0.61^a^^,^ b^,^ c	13.75±0.69^a^^,^ b^,^ c	2.33±0.13

NS, nonstress; Pb, lead; PS, prenatal stress.

3 point bending was applied to mouse femurs and resistance to force was calculated. Data are mean±s.e.m. for six mice per group.

a *P*<0.05 for effect of stress.

b *P*<0.05 for effect of Pb.

c *P*<0.05 for interaction of stress and Pb.

**Table 3:** Bone biomarkers and Wnt signaling molecules in serum of 8-month-old male mice

	0 Pb+NS	0 Pb+PS	100 Pb+NS	100 Pb+PS
CTx-1/(ng·mL^−1^)	56.02±2.39	68.27±5.85	61.88±8.12	66.30±3.94
P1NP/(ng·mL^−1^)	35.84±4.1	25.34±2.2^a^	23.15±3.31^b^	19.61±2.93^a^^,^ b
DKK-1/(pg·mL^−1^)	2.37±0.50	3.32±0.78	4.47±1.01	3.18±0.91
Sclerostin/(pg·mL^−1^)	332.69±58.57	366.46±56.84	507.81±70.72^b^	684.69±113.2^b^^,^ c

NS, nonstress; Pb, lead; PS, prenatal stress.

Systemic protein levels were measured using standard ELISA methods (*n*=5 per group) in trunk blood samples from stress- and Pb-treated mice.

a *P*<0.05 for effect of stress.

b *P*<0.05 for effect of Pb.

c *P*<0.05 for effect of Pb+stress.
